# ApInAPDB: a database of apoptosis-inducing anticancer peptides

**DOI:** 10.1038/s41598-022-25530-6

**Published:** 2022-12-09

**Authors:** Naser Faraji, Seyed Shahriar Arab, Alireza Doustmohammadi, Norelle L. Daly, Ahmad Yari Khosroushahi

**Affiliations:** 1 Department of Medical Nanotechnology, Faculty of Advanced Medical Sciences, Tabriz University of Medical Sciences, Tabriz, Iran; 2 Drug Applied Research Center, Tabriz University of Medical Sciences, Tabriz, Iran; 3 Department of Biophysics, Faculty of Biological Sciences, Tarbiat Modares University, Tehran, Iran; 4 Centre for Molecular Therapeutics, Australian Institute of Tropical Health and Medicine, James Cook University, Cairns, QLD Australia

**Keywords:** Cancer, Computational biology and bioinformatics, Drug discovery

## Abstract

ApInAPDB (Apoptosis-Inducing Anticancer Peptides Database) consists of 818 apoptosis-inducing anticancer peptides which are manually collected from research articles. The database provides scholars with peptide related information such as function, binding target and affinity, IC50 and etc. In addition, GRAVY (grand average of hydropathy), net charge at pH 7, hydrophobicity and other physicochemical properties are calculated and presented. Another category of information are structural information includes 3D modeling, secondary structure prediction and descriptors for QSAR (quantitative structure–activity relationship) modeling. In order to facilitate the browsing process, three types of user-friendly searching tools are provided: top categories browser, simple search and advanced search. Overall ApInAPDB as the first database presenting apoptosis-inducing anticancer peptides can be useful in the field of peptide design and especially cancer therapy. Researchers can freely access the database at http://bioinf.modares.ac.ir/software/ApInAPDB/.

## Introduction

**Figure 1:**
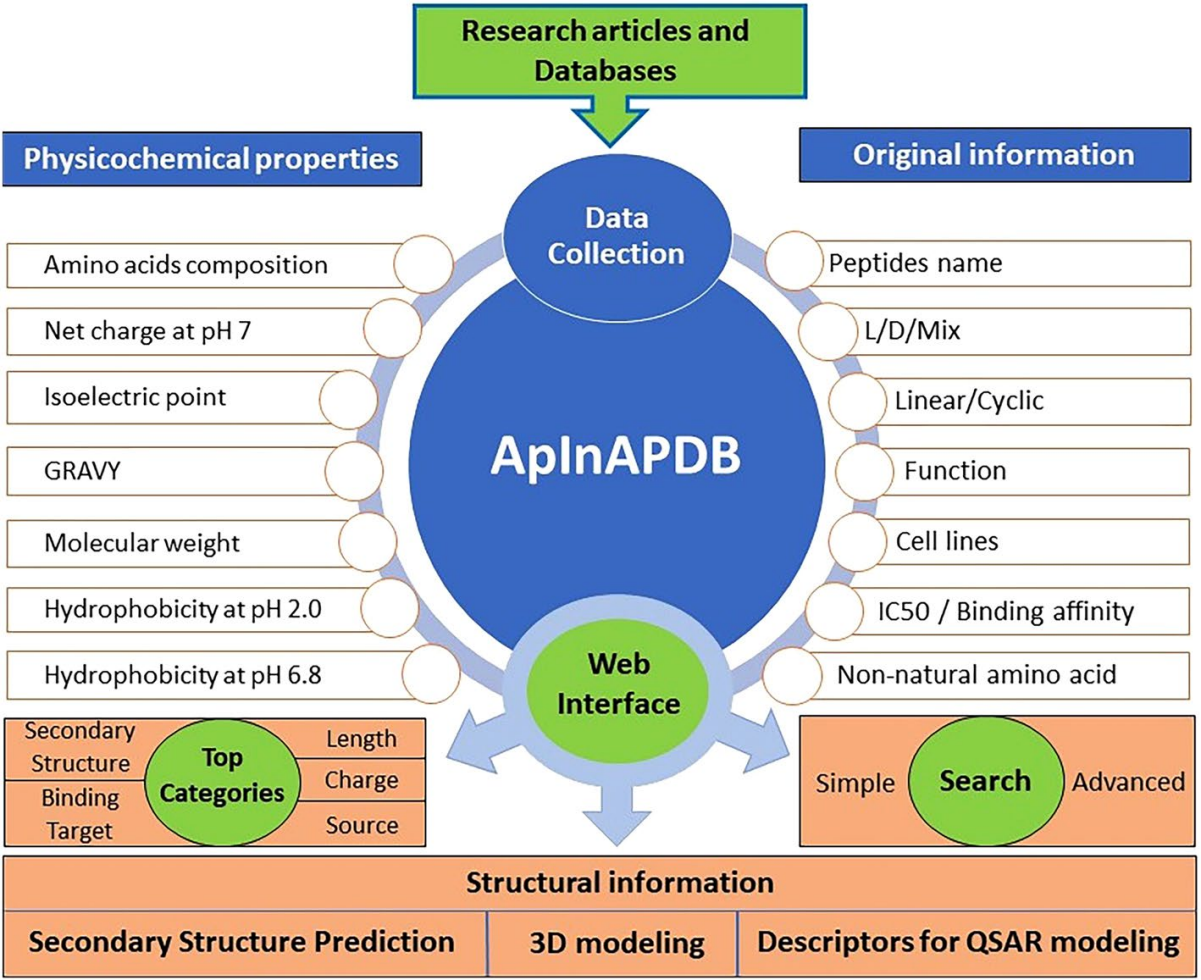
Overall structure and features of ApInAPDB.

Cancer, one of the most serious public health problems on a global scale^1^, has significant treatment challenges including multidrug resistance and a lack of tumor-specific therapies with minimal side effects^2^. Peptide-based therapy is a new strategy in cancer treatment^3^. Anticancer peptides (ACPs) possess some advantages like short sequence (less than 50 amino acids)^4^, easy synthesis and modification, cost-effectiveness, biocompatibility, specific targeting, and low intrinsic toxicity^4–6^ that makes them promising as anticancer therapeutics^2^. ACPs have been categorized based on structure and activity. The structural categories include α-helical, β-sheet, random coil, and cyclic peptides. Since α-helical and β-sheet conformations have their advantages and disadvantages in anticancer peptide performance^7^, secondary structure prediction plays an important role in anticancer peptide design. Although the anticancer mechanism of ACPs is not thoroughly understood^4^, they are generally divided into four groups based on their activities I) cell membrane disruption; II) apoptosis-inducing; III) anti-angiogenesis peptides, and IV) immune regulation^7^. Among these bioactivities, the apoptosis pathway is considered the most successful treatment in non-surgical cancer therapies because it induces minimal inflammation and damage into the treated tissues^8,9^, highlighting the potential of apoptosis-inducing peptides^10^. The BCL-2 family is the main proteins that regulate the intrinsic apoptosis pathway while TNFR, FAS (CD95), and DR3/WSL are involved in extrinsic apoptosis^11^. Peptide-based cancer therapy can target both apoptosis pathways^12,13^. However, the origin of apoptosis-inducing anticancer peptides is not confined to analogues of the BCL-2 family. Numerous peptides such as human second mitochondria-derived activator of caspase (SMAC) peptides, venom-derived peptides and also antimicrobial peptides have been discovered with apoptosis induction effects on cancerous cells^14–16^. Despite significant interest in peptide-based cancer therapy, there are limited anticancer peptide databases like CancerPPD^4^ (anticancer peptides and proteins database), and to our knowledge, no database specifically includes apoptosis-inducing peptides. Here we have established ApInAPDB (Apoptosis-Inducing Anticancer Peptides Database) as a comprehensive database that is dedicated to apoptosis-inducing peptides and their analogues regardless of their degree of effectiveness. The information available in the database, taken from the literature, includes binding target, function, and their effectiveness reported as IC50, binding affinity or spot array. This database also presents additional information such as amino acid composition, hydrophobicity, charge and also prediction of secondary structure using different algorithms. All properties calculated for each peptide and also different algorithms used for structural prediction will be discussed in the database information section. The overall ApInAPDB structure with its features is shown in Fig. 1. This database has the potential to aid in the development and design of novel apoptosis-inducing peptides.

## Results

**Figure 2:**
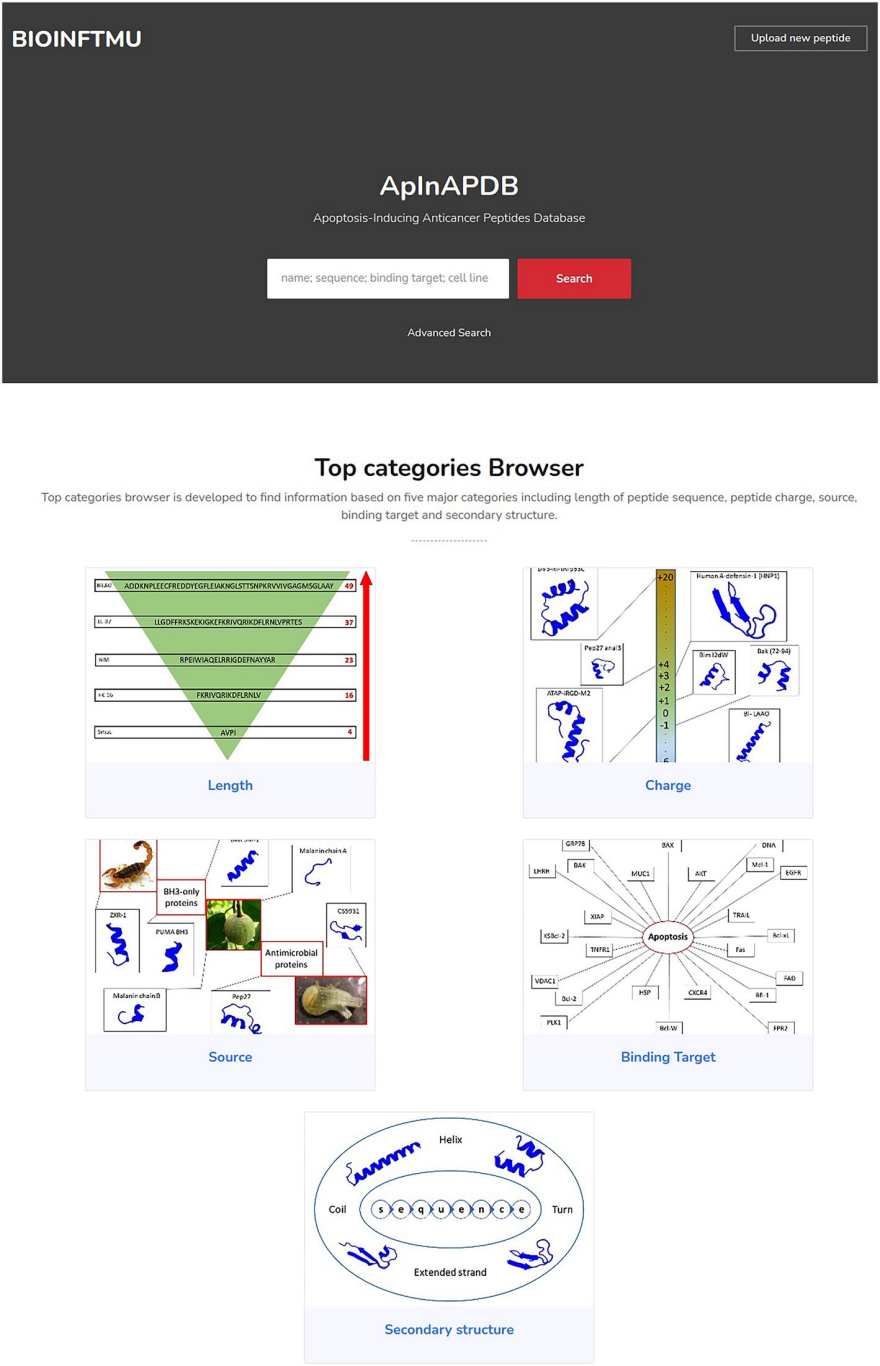
Schematic representation of ApInAPDB interface.

The ApInAPDB interface (Fig. 2) has been designed for searching and browsing the database conveniently. Instructions on how to search and browse in this user-friendly interface are given below.

### Searching tool

#### Simple search

Through a simple search box, keywords can be searched on peptide name, cell line, whole/partial peptide sequence or binding target. Here the keyword search for each field is done separately.

#### Advanced search

**Figure 3:**
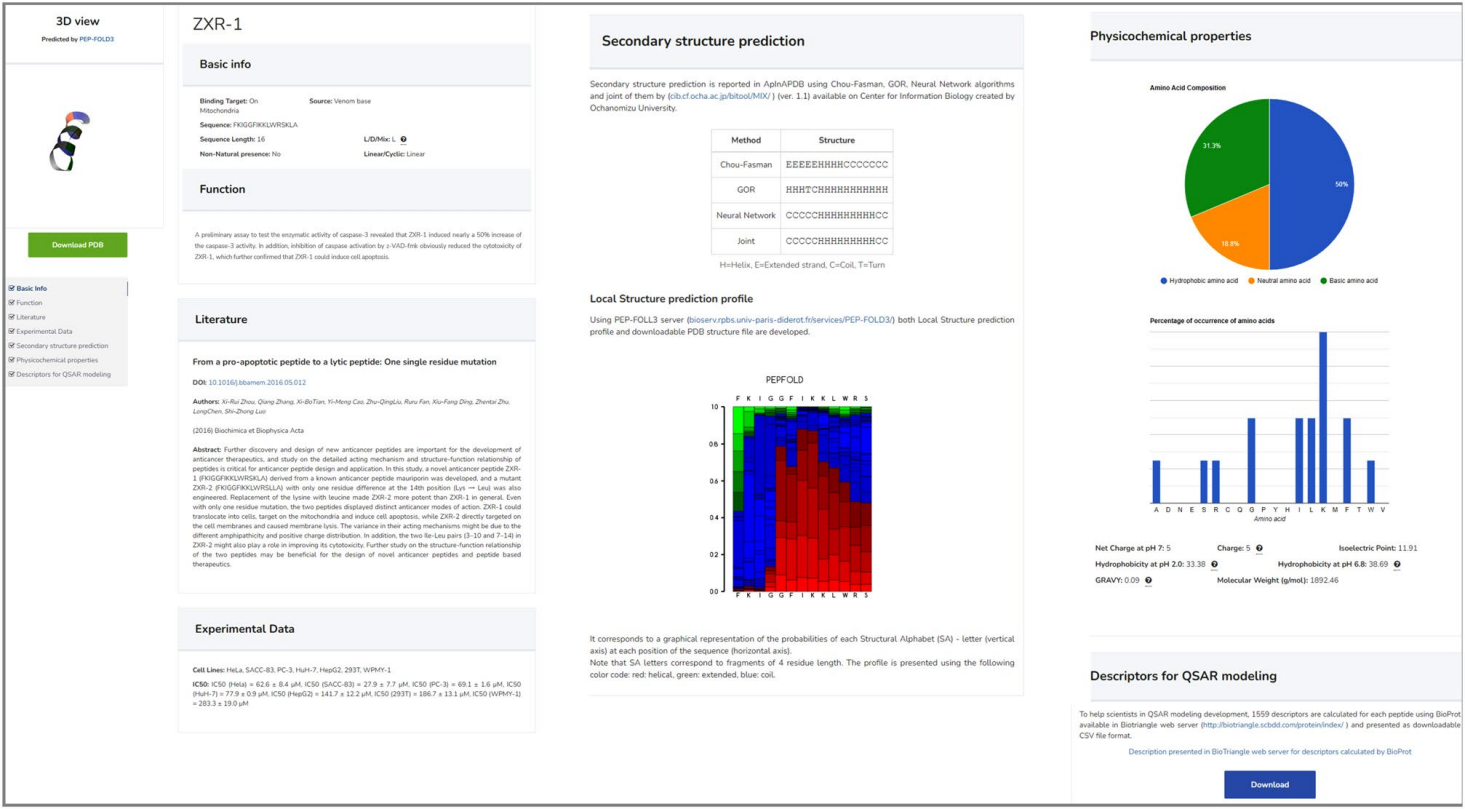
An example of result page: Original information (left), secondary structures prediction (middle), physicochemical properties (right top) and descriptors for QSAR modeling (right down).

This search allows the users to select more than one field at a time. For some fields such as charge of peptide, a range can be defined and for some fields such as cell line, more than one option can be selected. Consequently, users can find peptides with multiple desired properties. Among these properties, secondary structure is more complex and users can confine the searching via parameters including Min/Max content of Helix, Extended, Turn and random coil peptides. An example of result page is displayed in Fig. 3.

### Top categories browser

**Figure 4:**
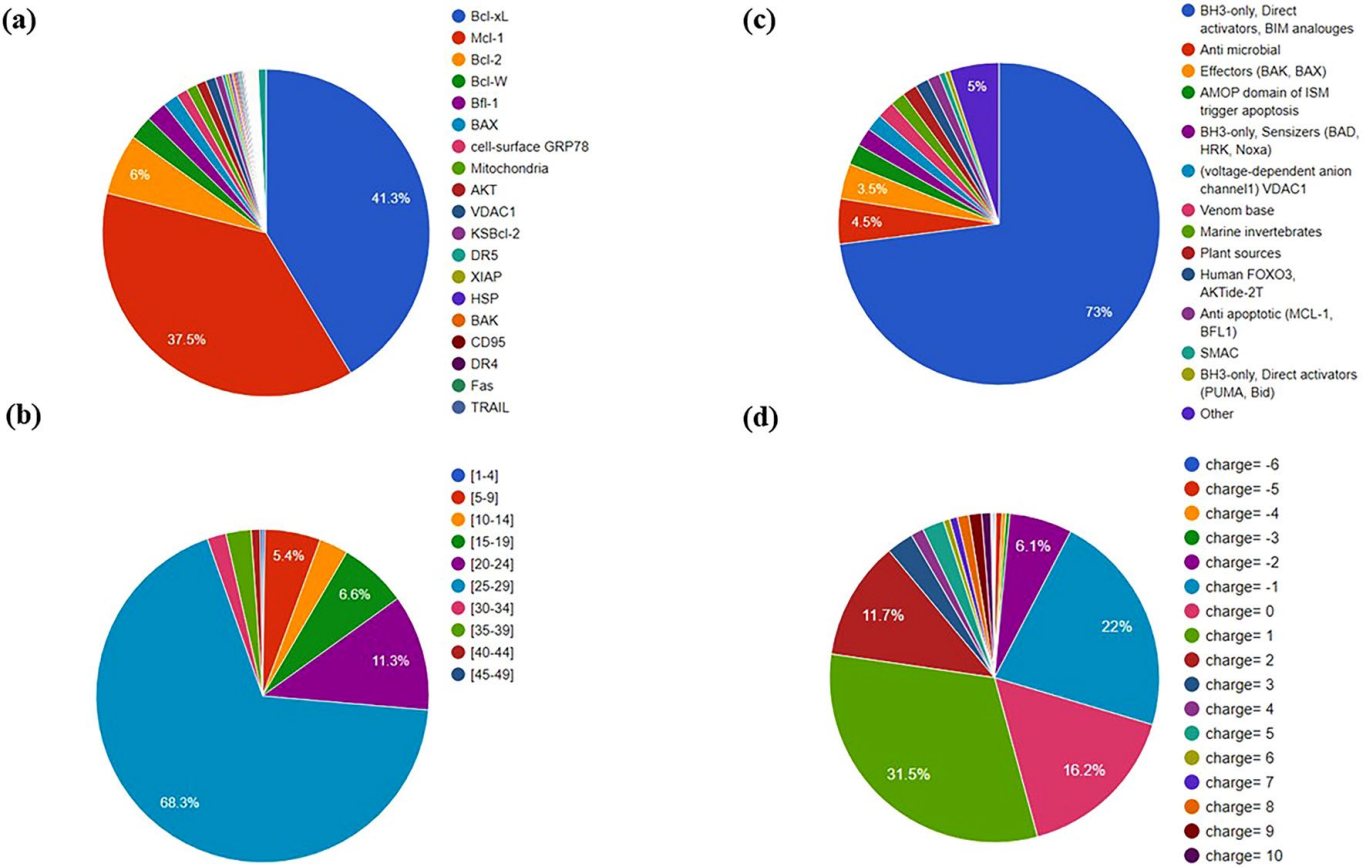
Representation of distribution based on (**a**) binding target, (**b**) length, (**c**) source and (**d**) charge in ApInAPDB.

The top categories browser was developed to find information based on five major categories including length of peptide sequence, peptide charge, source, binding target and finally secondary structure. Through this, users can browse the database more easily. As length, charge and secondary structure play crucial roles in peptide efficiency and knowing their source and binding target is critical information, users can browse and compare the different peptides based on these categories and gain more insight into peptide design. A schematic representation of distribution of apoptosis-inducing Anticancer Peptides based on length, binding target, source and charge is shown in Fig. 4.

### Data statistics

In total 818 peptides and analogues are listed in ApInAPDB; 787 linear and 31 cyclic peptides. The database consists of 797 peptides containing L-amino acids, one containing D-amino acids and 20 having both L and D amino acids. 224 peptides have been evaluated on different cell lines including HeLa, MCF-7, Jurkat, MDA-MB-435, HepG2, and for 119 peptides IC50 values are reported. The cell line and IC50 of each peptide are stored in the database. Binding affinity of 628 peptides are provided: for 110 peptides ki or kd and for 518 peptides, SPOT array substitution analysis binding to Mcl-1 (100 nM) and Bcl-xl (100 nM) are reported and compiled in the database. The secondary structures of 765 peptides were predicted using different algorithms available on Center for Information Biology (CIB) facilities. Cyclic peptides, D-peptides and those containing modified amino acids were not included in this prediction due to the limitation algorithms. Also, using a de novo approach with the PEP-FOLD3 server, peptide structures were predicted. Some peptides were not considered in this modeling because of limitations with PEP-FOLD3 including peptide size (between 5 and 50 residues) and amino acid types. In total, the structures of 765 peptides were predicted and then compiled in the database. In addition, descriptors of 777 peptides were calculated using Biotriangle web server for QSAR (quantitative structure–activity relationship) modeling development. Other peptides were excluded due to amino acid type's limitation of the web server.

## Discussion

Peptides possess considerable potential in cancer therapy due to their target specificity. Several peptides have recently been approved for cancer treatment, including leuprolide, Buserelin, Gonadorelin, Cetrorelix, Abarelix, Degarelix, and Octreotide. In addition, several anticancer peptides are in or about to enter clinical trials, including BT1718, DPT-C9h, p28^10,17^. Since deregulation of apoptosis is a feature of many cancers, drugs including peptides which induce normal apoptosis pathway are potential candidates for treating cancers efficiently^18^, Thus, there is great interest in designing apoptosis-inducing peptides for cancer therapy, and abundance of literature is available in this area^19,20^. There are several databases which collected peptides and proteins used in cancer therapy researches like CancerPPD^4^, TumorHope^21^ (tumor homing peptides database), dbPepNeo^22^ (human tumor neoantigen peptides database) and ApoCanD^23^ (human apoptotic proteins database on cancer) but there is not any database which present specifically apoptosis-inducing peptides thus this study aimed to develop ApInAPDB to cover this gap. ApInAPDB attempts to present information for researchers interested in studying and developing apoptosis inducing peptides in order to: (1) find the most efficient peptide, (2) determine if designed peptides are novel, (3) perform docking, molecular dynamic simulation and screening for peptides with known binding target reported for each peptide (4) develop QSAR models for those analogues with the same lead peptide/ protein using calculated descriptor files (5) design apoptosis-inducing peptides focusing on the secondary structure prediction, (6) find comprehensive physicochemical properties as well as original information, and (7) browse database with a user-friendly searching tool.

## Methods

### Data collection

This study aimed to establish a database including anticancer peptides with apoptosis activity regardless of their degree of effectiveness. To cover this aim, collecting and investigating a great number of research articles and available databases such as DRAMP^24^ (antimicrobial peptides database) are required. The most relevant research articles were manually gathered from Scopus, Google Scholar, and PubMed using keywords such as 'apoptotic peptides', 'BCL2 family peptides', 'apoptosis-inducing peptides', 'BH3 mimicking peptides', 'anticancer peptides' and their combination. The collected articles were carefully studied by the authors to select those that reported the apoptosis-inducing property for their investigated peptide. Accordingly, the irrelevant articles were excluded and relevant articles that experimentally recommended the apoptosis-inducing peptides or innately apoptotic peptides like Bim (BCL-2 interacting mediator of cell death) analogues were included in the database. Totally 818 apoptosis-inducing peptides were presented in the ApInAPDB. Out of them, 783 peptides were collected from research articles and 35 peptides were collected from DRAMP database.

### ApInAPDB web interface

Using MySQL (version 8.0), the collected data is managed. There are two parts to the development of the web interface: front-end development (what the user sees when he encounters the website) and back-end development (connecting the website to the database and querying it to get results). The front end of the website was developed using HTML5, CSS3, PHP (version 7.4), JavaScript ES6, and Jquery (version 3.5.1). The back-end of the website is also developed using PHP. In addition, Google Charts was used to plot all of the charts.

### Database information

Three categories of information are stored in ApInAPDB as original, physicochemical properties and structural information. The original information gathered from research articles includes peptides name, source, sequence, sequence length, N-terminal modification, C-terminal modification, binding target, L/D/Mix, presence of non-natural amino acid, linear/cyclic, function, journal name, publication year, DOI, authors, the title of the paper, cell lines, IC50 of peptide and binding affinity. The next category is physicochemical properties which are derived from the original information. Since peptide properties such as isoelectric point, molecular weight, net charge, etc., play an important role in cancer cell proliferation inhibition or eradication^25^, they are reported in the database including molecular weight (g/mol), amino acid composition, hydrophobic/acidic/neutral/basic amino acid percentage, net charge at pH 7, isoelectric point, hydrophobicity at pH 2.0, hydrophobicity at pH 6.8 and charge using biosyn peptide property calculator v3.1 online tool (https://www.biosyn.com). Also, GRAVY (grand average of hydropathy) of each peptide is reported in the database using novoprolabs peptide property calculator online tool (https://www.novoprolabs.com/tools/calc_peptide_property). The third and last category is structural information including descriptors for QSAR modeling, secondary structure prediction and 3D modeling. To help scientists in QSAR modeling development, 1559 descriptors are calculated for each peptide using BioProt available in Biotriangle web server (http://biotriangle.scbdd.com/protein/index/)^26^ and presented as downloadable CSV file format (The exemplary hyperlink belongs to ZXR-1 peptide). As peptide secondary structure plays an important role in binding to the target, secondary structure prediction is reported in ApInAPDB database using GOR (Garnier, Osguthorpe and Robson method), Neural Network and Chou-Fasman algorithms (http://cib.cf.ocha.ac.jp/bitool/MIX/) (ver. 1.1) available on Center for Information Biology (CIB) created by Ochanomizu University. Furthermore, CIB provides users with the facility to make a consensus on the secondary structure prediction according to the three aforementioned algorithms. Using this ability, a consentaneous result is reported for each peptide. Additionally, using PEP-FOLD3 server (https://bioserv.rpbs.univ-paris-diderot.fr/services/PEP-FOLD3/)^27^ both Local Structure prediction profile and downloadable PDB structure file are available (The exemplary hyperlink belongs to ZXR-1 peptide).

## Conclusions

Nowadays, apoptosis-inducing peptides play an important role in cancer therapy and they attract the attention of researchers in the field of peptide design and synthesis. This fact calls for a comprehensive database that includes this type of peptide as a reference for scholars. ApInAPDB is a database that consists of more than 800 apoptosis-inducing anticancer peptides collected from different articles and it provides comprehensive and useful information about each peptide. The information includes physicochemical properties, secondary structure prediction and descriptors for QSAR modeling among others. Since updating the database with novel information is very important, authors will attempt to update it and add newly studied apoptosis-inducing peptides with their original, physicochemical properties and structural information. Hence, ApInAPDB is certainly helpful for researchers willing to design apoptosis-inducing peptides for cancer therapy.

### Limitations

In the ApInAPDB database, structural information is not prepared for cyclic peptides and peptides containing modified amino acids due to the lack of online calculation and prediction tools. For D-peptides, only those physicochemical properties are reported which can be calculated with available online tools.

## Data Availability

This database is freely available through the following address. (http://bioinf.modares.ac.ir/software/ApInAPDB/).
